# High-Throughput Sequencing Analysis of the Actinobacterial Spatial Diversity in Moonmilk Deposits

**DOI:** 10.3390/antibiotics7020027

**Published:** 2018-03-21

**Authors:** Marta Maciejewska, Magdalena Całusińska, Luc Cornet, Delphine Adam, Igor S. Pessi, Sandrine Malchair, Philippe Delfosse, Denis Baurain, Hazel A. Barton, Monique Carnol, Sébastien Rigali

**Affiliations:** 1 InBioS—Centre for Protein Engineering, Institut de Chimie B6a, University of Liège, B-4000 Liège, Belgium; maciejewska.m@wp.pl (M.M.); delphine.adam@doct.ulg.ac.be (D.A.); ispessi@alumni.ulg.ac.be (I.S.P.); 2 Environmental Research and Innovation Department, Luxembourg Institute of Science and Technology, Belvaux, Luxembourg; magdalena.calusinska@list.lu (M.C.); philippe.delfosse@list.lu (P.D.); 3 InBioS—PhytoSYSTEMS, Eukaryotic Phylogenomics, University of Liège, B-4000 Liège, Belgium; luc.cornet@uliege.be (L.C.); Denis.Baurain@uliege.be (D.B.); 4 InBioS—Plant and Microbial Ecology, Botany B22, University of Liège, B-4000 Liège, Belgium; S.Malchair@uliege.be (S.M.); S.Malchair@uliege.be (M.C.); 5 Department of Biology, University of Akron, Akron, OH 44325, USA; bartonh@uakron.edu

**Keywords:** antibiotics, geomicrobiology, Illumina sequencing, microbiome diversity, *Streptomyces*, Actinobacteria

## Abstract

Moonmilk are cave carbonate deposits that host a rich microbiome, including antibiotic-producing Actinobacteria, making these speleothems appealing for bioprospecting. Here, we investigated the taxonomic profile of the actinobacterial community of three moonmilk deposits of the cave “Grotte des Collemboles” via high-throughput sequencing of 16S rRNA amplicons. Actinobacteria was the most common phylum after Proteobacteria, ranging from 9% to 23% of the total bacterial population. Next to actinobacterial operational taxonomic units (OTUs) attributed to uncultured organisms at the genus level (~44%), we identified 47 actinobacterial genera with *Rhodoccocus* (4 OTUs, 17%) and *Pseudonocardia* (9 OTUs, ~16%) as the most abundant in terms of the absolute number of sequences. Streptomycetes presented the highest diversity (19 OTUs, 3%), with most of the OTUs unlinked to the culturable *Streptomyces* strains that were previously isolated from the same deposits. Furthermore, 43% of the OTUs were shared between the three studied collection points, while 34% were exclusive to one deposit, indicating that distinct speleothems host their own population, despite their nearby localization. This important spatial diversity suggests that prospecting within different moonmilk deposits should result in the isolation of unique and novel Actinobacteria. These speleothems also host a wide range of non-streptomycetes antibiotic-producing genera, and should therefore be subjected to methodologies for isolating rare Actinobacteria.

## 1. Introduction

Molecular approaches evaluating microbial communities in caves have revealed a level of diversity greater than initially expected [1]. Microorganisms have been found to inhabit virtually all subterranean niches, including cave walls, ceilings, speleothems, soils, sediments, pools, and aquifers [2]. Cave bacteria often represent novel taxonomic groups [3,4,5,6,7], which are frequently more closely related to other cave-derived bacterial lineages than to the microbiota of other environments [8,9,10]. 

Among cave speleothems, moonmilk draws a particular scientific attention due to its distinctive crystal morphology. The origins of various moonmilk crystalline habits, including monocrystalline rods, polycrystalline chains, and nanofibers, are tentatively attributed to the moonmilk indigenous microbial population [11]. Among a moonmilk microbiome comprising Archaea, Fungi, and Bacteria [9,10,12,13,14,15,16,17,18,19], the indigenous filamentous Fungi [20] and Actinobacteria [11,21] are believed to mediate moonmilk genesis with cell surfaces promoting CaCO_3_ deposition [11,20,21]. Actinobacteria were additionally reported to be metabolically capable of inducing favorable conditions for CaCO_3_ precipitation, or even directly precipitating carbonate minerals [12,21]. Members of the phylum Actinobacteria are routinely found in this speleothem [9,10,12,13,14,18,19], as well as in the other subterranean deposits within limestone caves [3,8,22,23], volcanic caves [24,25,26], and ice caves [27]. The broad distribution of Actinobacteria in the subsurface systems stimulates investigation in order to understand the factors driving their existence in mainly inorganic and highly oligotrophic environments, and the processes that enable them to mediate speleogenesis. The successful adaptation of Actinobacteria to a wide range of environments could probably be a consequence of their broad-spectrum metabolism, which includes prolific secreted hydrolytic systems that are capable of generating nutrient sources from various substrates, along with their extraordinary faculty to produce specialized metabolites (metal chelators, antimicrobials, hormones, etc.) [28]. 

As recently reported, moonmilk Actinobacteria represent novel microorganisms, which is a discovery that opens great avenues for the bioprospecting of novel drugs [6,10,18]. Rooney et al. (2010) [13] showed that spatially separated moonmilk speleothems in Ballynamintra Cave are inhabited by taxonomically distinct fungal and bacterial communities. Instead, in our attempt to isolate moonmilk-dwelling Actinobacteria for assessing their potential for participating in the genesis of these speleothems [21] and producing antimicrobial compounds [10], we only recovered members of the genus *Streptomyces*. Such a dominance of streptomycetes was rather unexpected, according to other moonmilk microbial diversity studies performed through culture-dependent [10,12,13,18] and culture-independent approaches using clone libraries [9], denaturing gradient gel electrophoresis (DGGE) fingerprinting [14,16,17], automated ribosomal intergenic spacer analysis (ARISA) [13], and, more recently, high-throughput sequencing (HTS) [19]. The actinobacterial genera identified in those studies included *Rhodococcus*, *Pseudonocardia*, *Propionibacterium*, *Nocardia*, *Amycolatopsis*, *Saccharothrix*, *Geodermatophilus*, *Mycobacterium*, *Aeromicrobium*, *Kribella*, *Nocardioides*, *Actinomycetospora*, *Nonomuraea*, *Euzebya*, *Rubrobacter*, and *Arthrobacter*, in addition to *Streptomyces*. Nonetheless, the diversity of the moonmilk actinobacterial microbiome still remains largely unknown and, beyond evaluating “*what and how much have we missed in our culture-dependent bioprospecting approach*” [10], a major important question that arises is: *to what extent are moonmilk-dwelling Actinobacteria different between the moonmilk deposits within a single cave, or in different caves?*

In this work, we carried out a comparative (HTS) of 16S small subunit (SSU) rRNA gene from DNA extracted from spatially separated moonmilk deposits within the same cave, “Grotte des Collemboles” (Springtails’ Cave) in Comblain-au-Pont, Belgium (Figure S1), in order to draw a detailed taxonomic picture of the intra-phylum diversity. Identifying the presence of rare Actinobacteria and unveiling to which degree they exhibit a spatial variability would help determining whether it is worth prospecting from different moonmilk deposits to isolate unique and novel natural compound producers.

## 2. Results

### 2.1. Actinobacterial Abundance within the Whole Moonmilk Bacterial Microbiome

**Figure 1:**
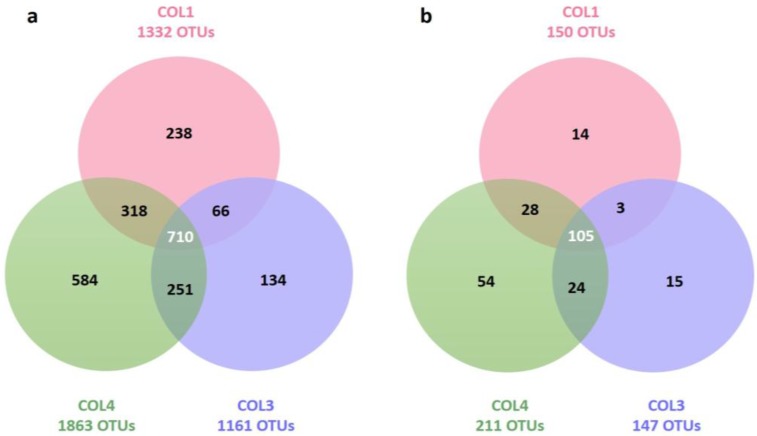
Venn diagrams showing the numbers of shared and unique bacterial (**a**) and actinobacterial (**b**) OTUs between the three moonmilk sampling points (COL1, COL3, COL4).

Libraries spanning the V4–V6 variable regions of the 16S rRNA gene using universal bacterial primers were used to assess the proportion of Actinobacteria in comparison to the whole bacterial community of three moonmilk deposits of the cave “Grotte des Collemboles” (Table S1a). The observed bacterial communities differed in species richness, evenness, and diversity between the three sampling points (Table 1). Phylotype richness (total number of operational taxonomic units (OTUs) per site) was the highest in COL4 (1863 OTUs), followed by COL1 and COL3, with 1332 and 1161 OTUs, respectively (Table 1, Figure 1a). Across the three sampling points, we found a total of 2301 different OTUs, amongst which 710 (31%) were common to all of the deposits (Figure 1a). Interestingly, pairwise comparison revealed highly similar percentages (~31.7 ± 0.53%) of shared bacterial OTUs between moonmilk deposits (Table 2, Figure 1a). A total of 956 OTUs (42%) were found to be exclusive to one sampling site, with COL4 having the highest number of unique bacterial phylotypes (584 OTUs), along with the most diverse bacterial population, as reflected by the highest diversity indices (Table 1, Figure 1a). 

**Figure 2:**
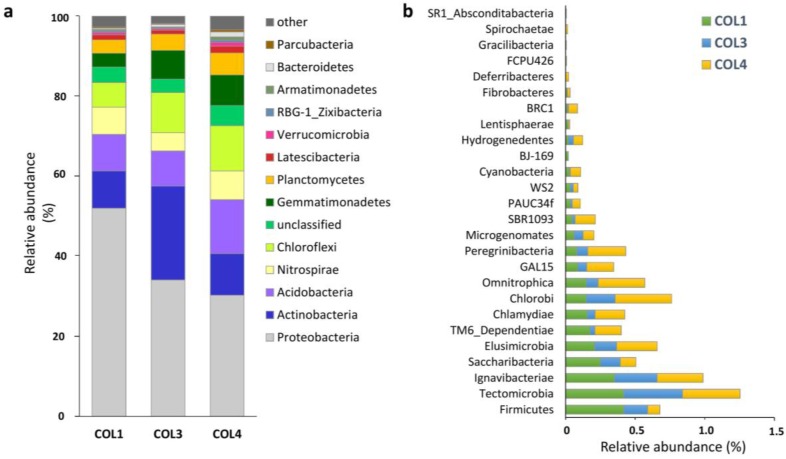
Taxonomic profiles of the moonmilk-associated microbiome at the phylum level across the three moonmilk sampling points (COL1, COL3, COL4). The main phyla of the microbiome are presented on the left (**a**), while the pattern of low-abundance taxa, named as ‘other’ (with a relative abundance of <1%) is displayed on the right (**b**).

Bacterial OTUs were grouped into 21 phyla and 18 candidate phyla (Table S2, Figure 2). Actinobacteria represented 9%, 23%, and 10% of the total bacterial population in COL1, COL3, and COL4, respectively (Table S2, Figure 2a). In terms of abundance, they were the most common phylum after Proteobacteria, which accounted for 52%, 34%, and 30% of the total community in COL1, COL3, and COL4, respectively (Table S2, Figure 2a). The other major phyla of the moonmilk microbiome included Acidobacteria, Nitrospirae, Chloroflexi, Gemmatimonadetes, Planctomycetes, Latescibacteria, Verrucomicrobia, Zixibacteria, Armatimonadetes, Bacteroidetes, and Parcubacteria (Table S2, Figure 2a). Together, these phyla constituted 93.4%, 94.7%, and 91.5% of the total community in COL1, COL3, and COL4, respectively (Table S2, Figure 2a). The remaining phyla (with a relative abundance of <1%) were pooled as ’other’ (Figure 2a), and included most of the candidate divisions identified in this study (Figure 2b). Sequences that could not be affiliated to any bacterial phylum accounted for 4%, 3%, and 5% of the sequences in COL1, COL3, and COL4, respectively (Table S2). Some fraction of the moonmilk microbial diversity still remains to be discovered for all of the three sampling sites, as the rarefaction curves did not reach a plateau (Figure S2a). 

### 2.2. Actinobacterial Diversity in Moonmilk Deposits

Evaluation of the actinobacterial profile was performed with libraries spanning the V6–V7 variable regions of 16S rRNA gene, and using modified Actinobacteria-specific primers (Table S1a). The specificity of the primers was confirmed by the detection of only 1%, 0.2%, and 2% of non-actinobacterial sequences in COL1, COL3, and COL4, respectively (Figure S3). In contrast to the bacterial dataset, the diversity of Actinobacteria appeared to be exhaustively sampled with the phylum-specific primers (Figure S2b). 

The diversity indices for Actinobacteria showed the same trends as the ones observed for the whole Bacteria domain, i.e., evenness and diversity were the highest in COL4, followed by COL3, and COL1 (Table 1). Phylotype richness was the highest in COL4 with 211 OTUs, followed by COL1 and COL3 with 150 OTUs and 147 OTUs, respectively (Figure 1b and Table 1). Among the 243 different OTUs, 105 OTUs (43%) were found in all three of the studied moonmilk deposits (Figure 1b). Hence, the moonmilk-associated actinobacterial community appeared to be more conservative than the moonmilk-associated bacterial population (31%, Figure 1a). If we also include OTUs shared between at least two sampling points, the level of conservation rises to 66% of OTUs for Actinobacteria, and 58% for Bacteria. Still, 34% of the 243 OTUs (14, 15, and 54 OTUs in COL1, COL3, and COL4, respectively) remained specific to a moonmilk deposit, despite the close localization of collection points within the studied cave (Figure 1b). COL4 was characterized not only with the highest number of unique phylotypes (54 OTUs) (Figure 1b), but also with the most diverse population, as revealed by diversity indices (Table 1). As observed for the bacterial dataset, pairwise comparisons showed highly similar percentages (~36.4% ± 0.41%) of shared actinobacterial OTUs between moonmilk deposits (Table 2). 

**Figure 3:**
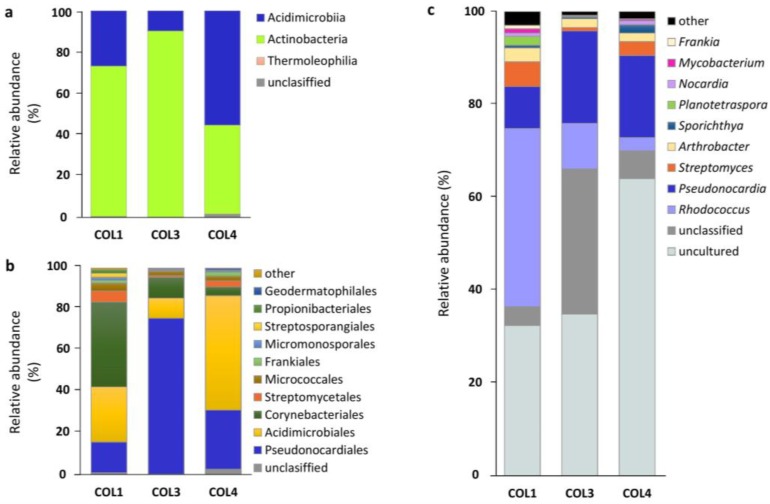
Taxonomic profiles of moonmilk-associated Actinobacteria at different taxonomic levels—(**a**) class; (**b**) order; (**c**) family—observed across the three moonmilk-sampling points (COL1, COL3, COL4). ‘Other’ includes orders and families with a relative abundance of <1%.

A taxonomic assignment of actinobacterial OTUs revealed the presence of two major classes—Acidimicrobiia and Actinobacteria, next to the low-abundant Thermoleophilia class (Table S3, Figure 3a). Acidimicrobiia was represented by one single order, the Acidimicrobiales, which dominated sample COL4, constituting 55.3% of the population (Table S3, Figure 3a). The Acidimicrobiales order consisted of two families, i.e., Acidimicrobiaceae and Iamiaceae (Table S3). The Actinobacteria class was represented by 15 orders, with Corynebacteriales dominating in COL1, and Pseudonocardiales in COL3 and COL4 (Table S3, Figure 3b). The most abundant families among the Actinobacteria class were Pseudonocardiaceae, Nocardiaceae, and Streptomycetaceae (Table S3). The proportion of unclassified and uncultured sequences at the family level ranged from 9% in COL3, to 25% in COL1, and 53% in COL4 (Table S3).

Among 28 families, a total of 47 genera were identified across the investigated samples (Table S3 and Table 3), with 35 genera identified for the first time in moonmilk (Table 3). COL1 was dominated by *Rhodococcus* (38.37%), while uncultured and unclassified Actinobacteria were the most abundant in COL3 and COL4 (Table 3). When only known genera were taken into account, *Pseudonocardia* prevailed in those samples, accounting for 20% and 18% of the population in COL3 and COL4, respectively (Table 3). Other genera, which constituted more than 1% of the population in at least one moonmilk deposit, included *Streptomyces*, *Arthrobacter*, *Sporichthya*, *Planotetraspora*, *Nocardia*, *Mycobacterium*, and *Frankia* (Table 3). While accounting in average for only 3% of the actinobacterial community, streptomycetes displayed the highest diversity, with 19 OTUs identified across the three moonmilk deposits (Table 3).

Some taxa showed important differences in their relative abundance between investigated samples, particularly *Rhodococcus*, which was approximately four and 14 times more abundant in COL1 than in COL3 and COL4, respectively (Table 3). The *Streptomyces* genus represented only 0.8% of the population in COL3, while it was detected at the level of 5.3% and 3% in the COL1 and COL4, respectively (Table 3). An important discrepancy in the relative abundance between speleothems was also observed for the genera *Planotetraspora*, *Mycobacterium*, and *Frankia*, whereas some taxa (e.g., *Pseudoclavibacter*, *Lentzea*, *Propionibacterium*) were exclusively found in a single sampling site (Table 3).

### 2.3. Analysis of the Most Abundant Actinobacterial OTUs

In order to obtain more information about the most dominant moonmilk-dwelling Actinobacteria, a detailed analysis was conducted for the 41 most abundant OTUs (~17% of all of the OTUs) accounting together for 90% (413,739 out of 456,878) of the sequences obtained via our HTS approach (Table 4). Out of the subset of 41 OTUs, 16 phylotypes belonged to the class Acidimicrobiia, with most of them being uncultured at the family level, and the remaining 25 OTUs belonged to the class Actinobacteria (Table 4). In the latter case, all of the OTUs were associated with major families previously identified in moonmilk deposits, including Pseudonocardiaceae, Propionibacteriaceae, Micrococcaceae, Nocardiaceae, Streptomycetaceae, and Streptosporangiaceae (Table 4). Only 16 OTUs could be classified at the genus level and were affiliated to genera *Rhodococcus*, *Pseudonocardia*, *Arthrobacter*, *Sporichthya*, *Streptomyces*, *Planotetraspora*, and *Nocardia* (Table 4).

Taking into account the spatial differences in terms of the most abundant taxa across the cave, COL1 was highly dominated by OTU1, affiliated to the genus *Rhodococcus*, and accounting for 38% of the total population in this speleothem (Table 4). This phylotype highly outnumbered other two *Rhodococcus* OTUs detected in COL1 (Table 3), which together constituted only 0.1% (data not shown). The predominant phylotypes identified in speleothems COL3 and COL4 were OTU2, representing an unclassified Pseudonocardiaceae in COL3 (29%), and OTU4, representing uncultured bacterium from Acidimicrobiia class in COL4 (11%) (Table 4). Among the known genera, *Rhodococcus* (OTU1, 10%) was also prevailing in COL3, while *Pseudonocardia* (OTU262, 6%) was found to be the most abundant in COL4 (Table 4). 

In total, 40 out of 41 OTUs were present in all the three studied moonmilk deposits, often with an extreme variation in terms of their relative abundance across the different collection points. This is well demonstrated by OTU2 (Pseudonocardiaceae, unclassified at the genus level), which largely dominated the actinobacterial community in COL3 (29%), while only representing 0.3% of the actinobacterial microbiome in COL1 (Table 4). 

### 2.4. Comparison of Moonmilk Streptomyces OTUs and Streptomyces Strains Isolated via the Culture-Dependent Approach

**Figure 4:**
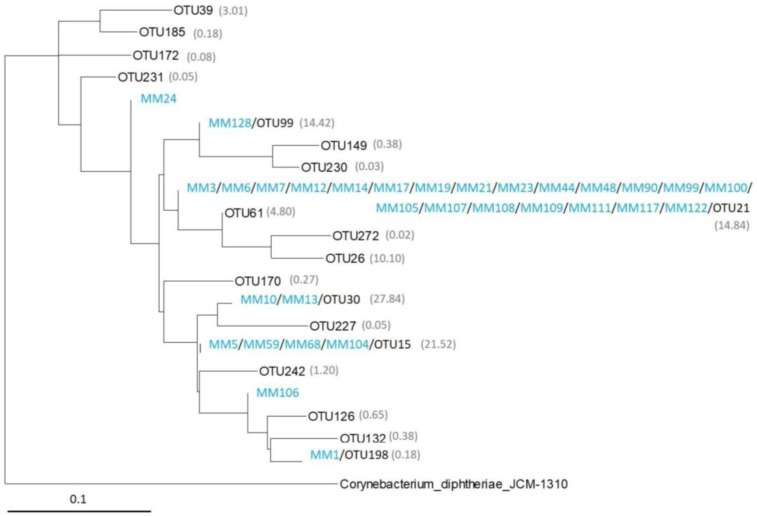
Phylogenetic relationships between culturable and non-culturable *Streptomyces* originating from moonmilk of “Grotte des Collemboles”. The tree was inferred by maximum likelihood. Scale bar is in substitution per site. Numbers between brackets reflect the predicted mean abundance of *Streptomyces* OTUs in the studied deposits based on the percentage of sequences retrieved from the HTS analysis. *Streptomyces* phylotypes isolated in our previous bioprospection study (MM strains) are marked in blue.

The true diversity of microbial communities is known to be strongly biased by cultivation-based methods in comparison to molecular techniques; therefore, we wanted to assess how much of the *Streptomyces* moonmilk-dwelling community we managed to isolate in our previous bioprospection work [10]. For this purpose, we compared the 16S rRNA sequences of the 19 *Streptomyces* OTUs retrieved from the HTS approach with the sequences of the 31 previously isolated *Streptomyces* phylotypes (MM strains), which were trimmed to the corresponding V6–V7 variable regions of HTS amplicons. Figure 4 presents the phylogenetic tree generated by maximum likelihood with all the 252 nt 16S rRNA sequences from the *Streptomyces* phylotypes (MM strains) and OTUs. The identity threshold for clustering sequences in the same branch of the tree was fixed to 97%, i.e., the same threshold as the one used to define OTUs in our HTS approach (see methods for details). As deduced from the generated phylogenetic tree, the 31 isolated *Streptomyces* strains matched with only five of the 19 *Streptomyces* OTUs, suggesting that the isolated strains represent a minor fraction of the *Streptomyces* species dwelling in the moonmilk deposits of the studied cave. Expectedly, Figure 4 further shows that we isolated *Streptomyces* species that are associated with the most abundant *Streptomyces* OTUs, e.g., OTU15, OTU21, OTU30, and OTU99 (Table 4), which together represent 79% of the *Streptomyces* sequences retrieved by our HTS approach. Moreover, 21 out of the 31 phylotype strains (68%) clustered together with OTU21 (Figure 4). Finally, two *Streptomyces* isolates, i.e., MM24 and MM106, did not cluster with any of the identified *Streptomyces* OTUs (Figure 4).

## 3. Discussion

### 3.1. New Insights into Moonmilk Bacterial Diversity Revealed by High-Throughput Sequencing

Previous investigations on the moonmilk microbiome revealed a very diverse microbial community in these deposits [9,13,14,15,16,17,19]. The high-throughput sequencing approach used in this work complemented previous findings by providing an in-depth picture of the bacterial population, together with a detailed taxonomic fingerprint of the phylum Actinobacteria. 

Comparison of the bacterial diversity in moonmilk between earlier investigations and the present work is limited to some extent by the differences in experimental procedures, such as DNA isolation and PCR-based approaches, and the sensitivity of the sequencing techniques. Nonetheless, the profile of the major taxonomic groups found in this work is consistent with that observed for the moonmilk communities in the caves “Grotta della Foos” and “Bus della Genziana” in Italy, which were obtained from 16S rRNA clone libraries [9]. All of the phyla detected in the above-mentioned caves, including Bacteroidetes, Acidobacteria, Chloroflexi, Planctomycetes, Verrucomicrobia, Actinobacteria, Firmicutes, Nitrospirae, Chlorobi, Proteobacteria, and WS3 (now Latescibacteria), were also identified in the cave “Grotte des Collemboles”, although their relative abundance varied between the studies. While Proteobacteria were found to be the most abundant phylum in both cases, the second most abundant population identified in Italian caves was the phylum Bacteroidetes, which constituted a minor part of the bacterial community in the present study. The Actinobacteria population was found to be an important part of the moonmilk microbiome in the “Grotte des Collemboles” (from 9% to 23%), but instead represented only a minor fraction (<2%) of the bacterial population in the two Italian caves investigated by Engel et al. (2013). Very recently, a study by Dhami et al. has reported the moonmilk microbiome profile in the Australian “Lake Cave” using an HTS approach [19], as in this work. The presence of Proteobacteria, Actinobacteria, Acidobacteria, Chloroflexi, Nitrospirae, Gemmatimonadetes, Firmicutes, and Bacteroidetes were detected in the moonmilk deposit of the “Lake Cave”, similarly to the “Grotte des Collemboles”. However, many of the low-abundance taxa identified in the Belgian cave were not reported, possibly because their phylogenetic profiles were based on different regions of 16S rRNA gene—V3/V4 for the “Lake Cave”, and V6–V7 for the “Grotte des Collemboles”. Interestingly, unlike in Italian and Belgian caves, the “Lake Cave” moonmilk deposit was strongly dominated by Actinobacteria, which were more than twice as abundant as the Proteobacteria [19]. The highly sensitive HTS amplicon sequencing approach employed in this work revealed the presence of 26 phyla within the moonmilk microbiome that had not been previously described in this speleothem. These included Zixibacteria (formerly RBG-1), Armatimonadetes (formerly OP10), and Parcubacteria (formerly OD1) among the main phyla of moonmilk microbiome (Figure 2a), which have been previously reported from other subterranean environments [24,29,30,31,32,33], and 23 low-abundant taxa that were found below the level of 1%, and included many candidate divisions (Figure 2b).

This new study uncovered a surprisingly diverse Actinobacteria taxonomic profile that demonstrates the limitations of our previous cultivation-based screening, in which only the *Streptomyces* species could be isolated from the three moonmilk deposits [10]. Here, a total of 47 actinobacterial genera from 28 families were identified across the investigated samples. Beyond the previously reported members of the Actinomycetales family—including *Nocardia* and *Rhodococcus* (Nocardiaceae) [15,18], *Pseudonocardia*, *Amycolatopsis* and *Saccharothrix* (not identified in our study) (Pseudonocardiaceae) [12,14,19], *Propionibacterium* (Propionibacteriaceae) [14], *Streptomyces* (Streptomycetaceae) [10,12,18,19], *Arthrobacter* (Micrococcaceae) [13], *Mycobacterium* (Mycobacteriaceae) [19], *Nocardioides*, *Aeromicrobium*, and *Kribbella* (Nocardioidaceae) [19], and *Geodermatophilus* (Geodermatophilaceae) [19]—35 other genera were identified in the moonmilk deposits of the “Grotte des Collemboles”. The population of each investigated sample was also found to include representatives of the Acidimicrobiia class, which were not previously reported in moonmilk. Their presence in all the three sampling sites, with an abundance up to 55% in COL4, and the dominance of the unclassified Acidimicrobiia phylotype (OTU 4) within the community of COL4, suggest that the chemical composition of the investigated moonmilk would be particularly suitable for the development of the representatives of this class of Actinobacteria, of which the ecology and metabolism are still largely unknown. 

### 3.2. Moonmilk Deposits as Appealing Source of Novel Producers of Bioactive Compounds

Extreme environmental niches have recently become the main targets for intense bioprospecting, as they are expected to host diverse yet-unknown microorganisms, which could offer unexplored chemical diversity. While *Streptomyces* are reported as the most prolific “antibiotic makers”, advances in the cultivation and characterization of rare Actinobacteria revealed similarly promising capabilities for the production of bioactive natural compounds [34,35,36]. The results obtained in this work suggest a significant biodiversity of the moonmilk-dwelling actinobacterial population, with a wide spectrum of rare genera. Next to *Streptomyces*, other members of Actinobacteria with valuable secondary metabolism were detected at a high proportion, such as *Pseudonocardia*, *Amycolatopsis*, *Streptosporangium*, *Nocardia*, *Nocardioides*, and *Rhodococcus*. Such findings clearly prompt to apply appropriate selective cultivation methods to isolate rare Actinobacteria from moonmilk deposits.

Moreover, particular importance should be also focused on Acidimicrobiia, which constituted an important part of the community in the studied deposits. Members of this class are a recently identified taxonomic unit [37] that is considered to represent an early-branching lineage within the phylum [38]. Due to their phylogenetic isolation and novelty, they are likely to hide a yet-uncovered valuable bioactive arsenal.

The great potential of moonmilk as a source of diverse and metabolically beneficial Actinobacteria is illustrated by the comparison of *Streptomyces* isolated in our previous study and the *Streptomyces* OTUs identified in this work (Figure 4). Most *Streptomyces* OTUs are phylogenetically distinct from culturable representatives (Figure 4), indicating that a great number of species still remain to be isolated. On the other hand, our culture-dependent study identified *Streptomyces* strains (MM24 and MM106, Figure 4) that were not associated with OTUs deduced from the HTS approach, confirming that both strategies are complementary, and should be used in parallel for microbial diversity assessment [39,40]. In addition, next to the identification methods themselves, our data suggests that the diversity level can be also biased by the identity threshold that is used for OTU definition. The tree revealed that a single OTU (OTU21, Figure 4) clustered together with most of the phylotypes deduced from MLSA (multilocus sequence analysis), each most likely representing a distinct species [10]. This indicates that the 97% sequence homology threshold applied to the comparative analysis of the V6–V7 regions of the 16S RNA gene largely underestimated the number of *Streptomyces* species dwelling in a studied environmental niche.

## 4. Materials and Methods 

### 4.1. Site description and Sampling

The cave “Grotte des Collemboles” (Springtails’ Cave), located in Comblain-au-Pont (GPS coordinates 50°28′41″ N, 5°36′35″ E), Belgium (Figure S1, Maciejewska et al. 2017 for full description), was formed in Visean limestone and has the shape of a 70-m long meander. White to brown–orange moonmilk deposits are found on the walls in the first narrow chamber located at the entrance of the cave, as well as in the narrow passages leading deeper into the cave (Figure S1). Moonmilk samples used for total DNA extractions were aseptically collected in January 2012 from three spatially separated locations along about a 20-m transect in the cave. Soft moonmilk speleothems were scratched with sterile scalpels into sterile Falcon tubes from the wall in the first chamber, adjacent to the cave entrance (COL4), and from the walls in a narrow passage after the first chamber (COL1, COL3) (Figure S1). COL4 was located approximately 6 m from COL1, and 20 m from COL3 (Figure S1). Samples were immediately transferred to the laboratory, freeze-dried on a VirTis Benchtop SLC Lyophilizer (SP Scientific, Warminster, PA, USA), and stored at −20 °C. 

### 4.2. Total DNA Extraction and 16S rRNA Gene Amplicon High-Throughput Sequencing

The metagenetic approach applied in this work was performed on DNA extracted from three moonmilk deposits (COL1, COL3, and COL4) originating from the “Grotte des Collemboles”. Environmental genomic DNA isolation was carried out from 200 mg of the freeze-dried moonmilk samples COL1, COL3, and COL4 (Figure S1), using the PowerClean Soil DNA kit (MoBio, Carlsbad, CA, USA), according to manufacturer’s instructions. The integrity of purified DNA was assessed by agarose gel electrophoresis (1% *w*/*v*), and the dsDNA concentration was evaluated by Qubit fluorometer (Invitrogen, Carlsbad, CA, USA). 

The 16S rRNA gene amplicon libraries were generated using bacterial (S-D-Bact-0517-a-S-17/S-D-Bact-1061-a-A-17 spanning V4–V6 region [41]) and actinobacterial (Com2xf/Ac1186r, spanning V6–V7 region [42]) specific primer pairs. The Illumina platform-compatible dual index paired-end approach was designed as previously described [43] (detailed description provided in the Table S1a). Each forward and reverse primer consisted of an Illumina-compatible forward/reverse primer overhang attached to the 5′ end. Additionally, a heterogeneity spacer of four degenerate nucleotides (Ns) was added to the forward primer, between the primer overhang and the locus-specific sequence. The Illumina barcodes and sequencing adapters were added during the subsequent cycle-limited amplification step using Nextera XT Index kit (Illumina, San Diego, CA, USA). Triplicated PCR reactions were performed for each sample in 25 µL of volume containing 2.5 µL of total DNA, 5 µL of each primer (1 µM), and 12.5 µL of 2× Q5 High-Fidelity Master Mix (New England Biolabs, Ipswich, MA, USA). Amplification conditions for each set of primers are listed in Table S1b. The triplicated amplicons were visualized on 3% agarose gel, pulled, purified with Agencourt AMPure XP beads (Beckman Coulter, Brea, CA, USA), and quantified with the Qubit HS dsDNA assay kit (Invitrogen, Carlsbad, CA,) before being processed for index ligation, using the Nextera XT Index kit (Illumina, San Diego, CA, USA). The PCR amplifications were performed with the same enzyme and cycling conditions as described above [43], with the total number of cycles reduced to eight, and an annealing temperature of 55 °C. The resulting amplicons were purified with the Agencourt AMPure XP magnetic beads (Beckman Coulter, CA, USA), quantified, and pooled in equimolar concentrations. The library concentration was quantified by qPCR using a Kappa SYBR FAST kit (Kapa Biosystems, Wilmington, MA, USA), and subsequently, the library was normalized to 4 nM, denaturated, and diluted to the final concentration of 8 p.m. The resulting pool was mixed with the PhiX control and subjected to 2 × 300 bp paired-end sequencing on Illumina MiSeq platform (Illumina, San Diego, CA, USA). Raw sequences were deposited in the NCBI Sequence Read Archive (SRA) database under the Bioproject PRJNA428798 with accession numbers SRX3540524–SRX3540529.

### 4.3. 16S rRNA Amplicon Analysis

16S rRNA amplicon analysis was based for both Bacteria and Actinobacteria on forward reads only, owing to the poor quality of reverse reads. Quality trimming (prohibiting mismatches and ambiguities, ensuring a minimum quality score of 20 and removing the four degenerate nucleotides from the 5′ end) was carried out using CLC Genomic Workbench (Qiagen, Hilden, Germany). USEARCH [44] was applied for length trimming (minimum length = 240 nt) and dereplication. Operational taxonomic units (OTUs) for both bacterial and actinobacterial datasets were defined using a 97% identity threshold on 16S rRNA sequences. OTUs were clustered using the UPARSE algorithm [45], and their taxonomic position was assigned by MOTHUR [46] with SILVA v128 database [47]. OTUs were further classified using BLASTN [48] analyses against a local mirror of NCBI nt database (downloaded on 9 August 2017), through manual and automatic analyses. For the automatic approach, a last common ancestor (LCA) classification was performed with a custom parser mimicking the MEGAN algorithm [49], which we developed for analyses of genome contamination (Cornet et al., 2017, under review). A maximum number of 100 hits per OTU were taken into account. To consider a BLASTN hit, the E-value threshold was set at 1e-15, the minimum identity threshold was set at 95.5%, the minimum bit score was set at 200, and the bit score percentage threshold was set at 99% of the best hit. These thresholds were defined through preliminary analyses (data not shown). When the BLASTN hits are too numerous, the MEGAN-like algorithm frequently yields high-ranking LCAs (e.g., Bacteria) that are not informative in practice. In order to minimize this effect, we decided to skip uncultured/unclassified hits whenever other, more informative, hits also passed the thresholds. Moreover, when computing LCAs, we only considered the most frequent taxa, provided that they represented ≥95% of the (up to 100) accumulated BLASTN hits, so as to avoid uninformative classifications due to a few (possibly aberrant) outliers.

Normalized OTU abundance data was used to calculate α-diversity and β-diversity estimators using MOTHUR [46]. Community richness, evenness, diversity, and differential OTU abundance between samples were calculated using sobs, the Simpson index, the inverse Simpson index and Venn diagrams, respectively.

The 19 OTUs identified as *Streptomyces* were combined to 31 sequences (16S rRNA region V6–V7) from previously isolated *Streptomyces* phylotypes (MM strains) and dereplicated with the UCLUST algorithm [44] using an identity threshold of 97%. This yielded 21 clusters, to which we added the homologous region of *Corynebacterium diphtheriae* JCM-1310 as an outgroup. A multiple sequence alignment was built with MUSCLE [50] (default parameters), and then analyzed with PhyML [51] under a K80 + Γ_4_ model. Due to the limited amount of phylogenetic signal (short sequences from very related organisms), the resolution of the tree was low (bootstrap proportions <50 for nearly all nodes; data not shown).

## 5. Conclusions

Before the advent of metagenomics, bioprospecting was carried out blindly, with poor knowledge on the real potential of an ecological niche mined for novel organisms, enzymes, or bioactive compounds. The results of the metagenetic study presented here confirmed that different moonmilk deposits host their own indigenous microbial population, and thus each individual speleothem can be a source of a great biodiversity. Consequently, the observed important differences in the spatial diversity of Actinobacteria imply that bioprospecting within different moonmilk deposits—from different caves or within the same cave—could result in the isolation of unique and novel natural compound producers. Our study also revealed how many and which actinobacterial genera have been missed in our first attempt to isolate antibiotic producers. We now know that the *Streptomyces* strains of our collection isolated from the moonmilk deposits of the cave ‘Grotte des Collemboles’ [10] are just the tip of the iceberg. These results prompted us to apply a series of ‘*tips and tricks*’ to isolate other *Streptomyces* and representatives of other antibiotic-producing Actinobacteria that are present in different proportions in each moonmilk deposit. The results of our adapted protocols for the isolation of rare Actinobacteria are presented in the article ‘*Isolation*, *Characterization*, *and Antibacterial Activity of Hard-to-Culture Actinobacteria from Cave Moonmilk Deposits*’, which is published in the same special issue [52].

## Figures and Tables

**Table 1:** Richness, specificity, diversity, and evenness of the bacterial and actinobacterial communities in the three moonmilk deposits of the “Grotte des Collemboles”. OTUs: operational taxonomic units.

Target group	Site	Total OTUs (Richness)	Unique OTUs (Specificity)	Inverse Simpson Index (Diversity)	Simpson Index (Evenness)
**Bacteria**	**COL1**	1332	238 (17.9%)	13.23	0.01
	**COL3**	1161	134 (11.6%)	58.94	0.05
	**COL4**	1863	584 (31.3%)	155.31	0.08
**Actinobacteria**	**COL1**	150	14 (9.3%)	6.21	0.04
	**COL3**	147	15 (10.2%)	7.74	0.05
	**COL4**	211	54 (25.6%)	24.13	0.11

**Table 2:** Pairwise comparisons of shared OTUs between the moonmilk deposits.

Target group	COL1 and COL3	COL1 and COL4	COL4 and COL3
Bacteria	776/2493 (31.1%)	1028/3195 (32.2%)	961/3024 (31.8%)
Actinobacteria	108/297 (36.4%)	133/361 (36.9%)	129/358 (36.0%)

**Table 3:** Actinobacterial genera pattern in moonmilk deposits of the “Grotte des Collemboles” based on 16S rRNA amplicon libraries.

Genus	COL1			COL3			COL4			TOTAL		
	Seq.	%	OTUs	Seq.	%	OTUs	Seq.	%	OTUs	Seq.	Av. %	Diff. OTUs
**Uncultured**	46,346	32.38	65	58,903	34.80	63	92,444	63.98	90	197,693	43.7	96
***Rhodococcus***	54,920	38.37	3	16,636	9.83	3	4102	2.84	3	75,658	17.0	4
***Pseudonocardia* †**	12,913	9.02	7	33,640	19.87	8	25,545	17.68	9	72,098	15.5	9
**Unclassified**	5762	4.03	18	52,908	31.26	20	8668	6.00	26	67,338	13.8	32
***Streptomyces* †**	7628	5.33	10	1292	0.76	11	4347	3.01	18	13,267	3.0	19
***Arthrobacter***	4323	3.02	4	3214	1.90	4	2649	1.83	5	10,186	2.3	5
*Sporichthya* *	771	0.54	1	556	0.33	2	2515	1.74	2	3842	0.9	2
*Planotetraspora* *	2769	1.93	1	294	0.17	1	113	0.08	1	3176	0.7	1
***Nocardia***	1023	0.71	2	157	0.09	2	1212	0.84	2	2392	0.5	2
***Mycobacterium* †**	1299	0.91	2	127	0.08	2	440	0.30	3	1866	0.4	3
*Frankia* *	1067	0.75	2	77	0.05	1	126	0.09	3	1270	0.3	3
*Luedemannella* *	555	0.39	2	212	0.13	2	276	0.19	2	1043	0.2	2
*Longispora* *	371	0.26	1	374	0.22	1	110	0.08	1	855	0.2	1
*Agromyces* *	310	0.22	2	136	0.08	2	355	0.25	2	801	0.2	2
*Actinoplanes* *	591	0.41	2	85	0.05	2	111	0.08	2	787	0.2	2
*Nakamurella* *	416	0.29	1	114	0.07	1	101	0.07	1	631	0.1	1
***Nocardioides* †**	360	0.25	4	52	0.03	3	158	0.11	8	570	0.1	9
***Geodermatophilus* †**	78	0.05	1	70	0.04	2	374	0.26	2	522	0.1	2
*Catellatospora* *	95	0.07	3	70	0.04	2	141	0.10	4	306	0.07	4
***Kribbella* †**	120	0.08	1	36	0.02	1	135	0.09	2	291	0.07	2
*Kocuria* *	261	0.18	1	23	0.01	1	-	-	-	284	0.1	2
*Actinomyces* *	247	0.17	5	1	0.001	1	6	0.004	1	254	0.06	5
*Corynebacterium* *	151	0.11	1	32	0.02	3	10	0.01	2	193	0.04	5
*Rhizocola* *	-	-	-	27	0.02	1	145	0.10	1	172	0.06	1
*Microbacterium* *	108	0.08	1	48	0.03	1	5	0.003	1	161	0.04	1
*Iamia* *	107	0.07	1	-	-	-	31	0.02	1	138	0.05	1
*Pseudoclavibacter* *	138	0.10	1	-	-	-	-	-	-	138	0.1	1
*Lentzea* *	-	-	-	-	-	-	111	0.08	1	111	0.08	1
***Aeromicrobium* †**	73	0.05	1	-	-	-	28	0.02	2	101	0.04	2
***Amycolatopsis***	86	0.06	1	-	-	-	5	0.003	1	91	0.03	2
*Cryptosporangium* *	-	-	-	71	0.04	1	10	0.01	1	81	0.02	1
*Glycomyces* *	35	0.02	1	-	-	-	45	0.03	1	80	0.03	1
*Streptosporangium* *	61	0.04	1	-	-	-	19	0.01	1	80	0.03	1
*Smaragdicoccus* *	43	0.03	1	-	-	-	34	0.02	1	77	0.03	1
***Propionibacterium***	57	0.04	1	-	-	-	-	-	0	57	0.04	1
*Kineosporia* *	44	0.03	1	-	-	-	8	0.01	1	52	0.02	1
*Jatrophihabitans* *	-	-	-	21	0.01	1	24	0.02	1	45	0.01	1
*Promicromonospora* *	-	-	-	22	0.01	1	21	0.01	1	43	0.01	2
*Millisia* *	-	-	-	22	0.01	1	6	0.004	1	28	0.01	1
*Rothia* *	2	0.001	1	13	0.01	1	7	0.005	1	22	0.005	1
*Tessaracoccus* *	-	-	-	17	0.01	1	-	-	-	17	0.01	1
*Acidothermus* *	-	-	-	-	-	-	16	0.01	1	16	0.01	1
*Marmoricola* *	-	-	-	-	-	-	14	0.01	2	14	0.01	2
*Dermacoccus* *	-	-	-	-	-	-	11	0.008	1	11	0.01	1
*Ponticoccus* *	-	-	-	8	0.005	1	-	-	-	8	0.005	1
*Stackebrandtia* *	-	-	-	-	-	-	8	0.006	1	8	0.01	1
*Umezawaea* *	-	-	-	-	-	-	2	0.001	1	2	0.001	1
*Actinospica* *	-	-	-	-	-	-	1	0.001	1	1	0.001	1
*Propionimicrobium* *	-	-	-	-	-	-	1	0.001	1	1	0.001	1

For each taxon, the number of obtained sequences (Seq.) and their relative abundance (%), together with the number of OTUs, are given. The total number of sequences, average relative abundance, and total number of different OTUs obtained per genus are shown in the last three columns. Taxa marked with an asterisk (*) were reported for the first time in moonmilk deposits in this studyTaxa marked with a cross (†) were detected in moonmilk deposits in this work, and in the high-throughput sequencing (HTS)-based study of Dhami et al. [19]. Taxa underlined represent the ones that were also detected in other moonmilk microbial diversity studies [12,13,14,15,18]. Cases filled in grey highlight the most abundant genera in each studied sampling point. Abbreviations: Seq.—number of sequences identified.

**Table 4:** The relative abundance (%) and taxonomy assignment of the most abundant actinobacterial OTUs found across moonmilk samples within the “Grotte des Collemboles”.

OTU	COL1	COL3	COL4	Av. %	Class	Family	Genus
OTU1	38.28	9.68	2.74	16.90	Actinobacteria	Nocardiaceae	*Rhodococcus*
OTU2	0.31	28.89	2.13	10.44	Actinobacteria	Pseudonocardiaceae	unclassified
OTU8	0.75	13.96	0.58	5.10	Actinobacteria	Pseudonocardiaceae	uncultured
OTU4	3.35	1.13	11.47	5.32	Acidimicrobiia	uncultured	uncultured
OTU3	0.52	7.80	4.87	4.40	Actinobacteria	Pseudonocardiaceae	*Pseudonocardia*
OTU262	3.45	4.12	5.89	4.49	Actinobacteria	Pseudonocardiaceae	*Pseudonocardia*
OTU6	7.53	1.00	3.98	4.17	Acidimicrobiia	uncultured	uncultured
OTU12	2.97	6.61	1.82	3.80	Actinobacteria	Pseudonocardiaceae	uncultured
OTU5	3.53	1.70	5.87	3.70	Acidimicrobiia	uncultured	uncultured
OTU13	0.46	4.91	3.65	3.01	Actinobacteria	Pseudonocardiaceae	*Pseudonocardia*
OTU98	0.99	1.07	6.94	3.00	Acidimicrobiia	uncultured	uncultured
OTU203	1.73	0.31	5.92	2.66	Acidimicrobiia	uncultured	uncultured
OTU432	3.68	1.40	1.66	2.25	Actinobacteria	Pseudonocardiaceae	*Pseudonocardia*
OTU142	0.72	1.91	2.35	1.66	Actinobacteria	Pseudonocardiaceae	unclassified
OTU7	0	1.70	3.31	1.67	Actinobacteria	Pseudonocardiaceae	uncultured
OTU19	2.06	0.52	1.65	1.41	Actinobacteria	Micrococcaceae	*Arthrobacter*
OTU190	0.46	0.31	2.84	1.20	Acidimicrobiia	uncultured	uncultured
OTU10	0.81	0.29	1.68	0.93	Acidimicrobiia	Acidimicrobiaceae	uncultured
OTU9	0.24	0.15	2.39	0.93	Acidimicrobiia	uncultured	uncultured
OTU251	0.77	0.65	1.12	0.85	Actinobacteria	Pseudonocardiaceae	*Pseudonocardia*
OTU14	0.54	0.31	1.73	0.86	Actinobacteria	Sporichthyaceae	*Sporichthya*
OTU30	2.11	0.13	0.31	0.85	Actinobacteria	Streptomycetaceae	*Streptomyces*
OTU360	0.40	0.17	1.68	0.75	Acidimicrobiia	uncultured	uncultured
OTU24	1.93	0.17	0.08	0.73	Actinobacteria	Streptosporangiaceae	*Planotetraspora*
OTU11	0.38	0.03	1.72	0.71	Acidimicrobiia	uncultured	uncultured
OTU20	0.71	0.47	0.81	0.67	Acidimicrobiia	Iamiaceae	uncultured
OTU47	0.75	0.26	0.98	0.66	Acidimicrobiia	uncultured	uncultured
OTU15	0.30	0.17	1.48	0.65	Actinobacteria	Streptomycetaceae	*Streptomyces*
OTU192	0.01	1.65	0.01	0.56	Actinobacteria	Pseudonocardiaceae	uncultured
OTU16	0.24	0.51	1.11	0.62	Acidimicrobiia	uncultured	uncultured
OTU50	0.88	0.54	0.37	0.60	Actinobacteria	Pseudonocardiaceae	uncultured
OTU22	1.61	0.16	0.06	0.61	Actinobacteria	Propionibacteriaceae	unclassified
OTU23	0.27	1.19	0.10	0.52	Actinobacteria	Micrococcaceae	*Arthrobacter*
OTU54	0.13	0.92	0.46	0.50	Actinobacteria	Pseudonocardiaceae	*Pseudonocardia*
OTU18	0.48	0.12	1.04	0.54	Acidimicrobiia	uncultured	uncultured
OTU25	0.56	0.09	0.83	0.49	Actinobacteria	Nocardiaceae	*Nocardia*
OTU21	0.80	0.19	0.34	0.45	Actinobacteria	Streptomycetaceae	*Streptomyces*
OTU99	1.05	0.09	0.17	0.44	Actinobacteria	Streptomycetaceae	*Streptomyces*
OTU36	0.22	0.04	0.96	0.41	Acidimicrobiia	uncultured	uncultured
OTU44	0.80	0.14	0.26	0.40	Acidimicrobiia	uncultured	uncultured
OTU32	0.22	0.03	0.92	0.39	Actinobacteria	unclassified	unclassified

## Supplementary Materials

The following are available online at http://www.mdpi.com/2079-6382/7/2/27/s1, Figure S1: Localization of the “Grotte des Collemboles” (Springtails’ Cave) together with the cave map and visualization of the moonmilk deposit sampling points, Figure S2: Rarefaction curves of OTUs clustered at 97% sequence identity across the three moonmilk-sampling points for Bacteria (a) and Actinobacteria (b), Figure S3: Taxonomic profile of bacterial phyla generated with Actinobacteria-specific primers. Note the high specificity of Actinobacteria primers, Table S1: Details of the PCR primers used for community profiling of moonmilk samples (a) and—PCR conditions used for 16S rRNA amplification from moonmilk samples (b), Table S2: Relative abundance (%) of bacterial phyla identified in the three moonmilk deposits in “Grotte des Collemboles”. Low-abundant taxa with relative abundance <1% are marked in red, Table S3: Relative abundance (%) of the phylum Actinobacteria at different taxonomic levels identified in the three moonmilk deposits in the “Grotte des Collemboles”.
