# Supplementary material for: High-Throughput Sequencing Analysis of the Actinobacterial Spatial Diversity in Moonmilk Deposits
Source: Antibiotics (Basel). 2018 Mar 21;7(2):27. doi: 10.3390/antibiotics7020027 (PMC6023079; doi:10.3390/antibiotics7020027)
Supplement: Supplementary file 1 [file antibiotics-07-00027-s001.zip › Supplementary Tables.pdf]

Table S1. Details of the PCR primers used for community profiling of moonmilk samples **(a)** and - PCR conditions used for 16S rRNA amplification from moonmilk samples (**b)**.

a)

**Target organism**	**Primer name**	**Adapter sequence ***	**Primer sequence**	**Variable region**	**Size of amplicon**	**Reference**
**Bacteria**	515F (BcF)	TCGTCGGCAGCGTCAGATGTGTATAAGAG	5′-GCCAGCAGCCGCGGTAA- 3′	V4 - V6	~550 nt	[29]
	1061R (BcR)	GTCTCGTGGGCTCGGAGATGTGTATAAGA	5′-CRRCACGAGCTGACGAC- 3′			
**Actinobacteria**	Com2xf (ActF)	TCGTCGGCAGCGTCAGATGTGTATAAGAG	5′-AAACTCAAAGGAATTGACGG- 3′	V6 - V7	~270 nt	[30]
	Ac1186r (ActR)	GTCTCGTGGGCTCGGAGATGTGTATAAGA	5′-CTTCCTCCGAGTTGACCC- 3′			

*overhanging adapters were added at 5′-end before specific primer sequence

b)

**Target organism**	**Predenaturation**	**Denaturation**	**Anealing**	**Extension**	**Final extension**	**Nr of cycles**
**Bacteria**	98°C - 30s	98°C - 50s	54°C - 30s	72°C - 30s	72°C - 2min	22
**Actinobacteria**			55°C - 30s			26

Table S2. Relative abundance (%) of bacterial phyla identified in the three moonmilk deposits in "Grotte des Collemboles". Low-abundant taxa with relative abundance <1% are marked in red.

**Bacterial phyla**	**COL1**	**COL3**	**COL4**	**Mean**	**Standard deviation**
Proteobacteria	52.0	34.1	30.3	38.8	11.6
Actinobacteria	9.3	23.4	10.4	14.4	7.9
Acidobacteria	9.1	8.8	13.5	10.5	2.6
Chloroflexi	6.2	10.0	11.3	9.2	2.7
Nitrospirae	6.8	4.6	7.2	6.2	1.4
Gemmatimonadetes	3.4	7.1	7.6	6.1	2.3
Planctomycetes	3.4	4.1	5.5	4.3	1.1
Latescibacteria	1.3	0.9	1.6	1.3	0.4
Bacteroidetes	0.3	0.7	1.3	0.8	0.5
Verrucomicrobia	0.5	0.4	0.9	0.6	0.3
Armatimonadetes	0.4	0.2	0.8	0.5	0.3
Tectomicrobia	0.4	0.4	0.4	0.4	0.0
RBG-1_Zixibacteria	0.4	0.2	0.5	0.4	0.2
Parcubacteria	0.3	0.2	0.5	0.3	0.2
Ignavibacteriae	0.4	0.3	0.3	0.3	0.0
Chlorobi	0.1	0.2	0.4	0.3	0.1
Firmicutes	0.4	0.2	0.1	0.2	0.2
Elusimicrobia	0.2	0.2	0.3	0.2	0.1
Omnitrophica	0.1	0.1	0.3	0.2	0.1
Saccharibacteria	0.2	0.1	0.1	0.2	0.1
Peregrinibacteria	0.1	0.1	0.3	0.1	0.1
Chlamydiae	0.2	0.1	0.2	0.1	0.1
TM6_Dependentiae	0.2	0.04	0.2	0.1	0.1
GAL15	0.1	0.1	0.2	0.1	0.1
SBR1093	0.05	0.02	0.1	0.1	0.1
Microgenomates	0.1	0.1	0.1	0.1	0.01
Hydrogenedentes	0.02	0.04	0.1	0.04	0.02
Cyanobacteria	0.03	0.01	0.1	0.04	0.03
PAUC34f	0.03	0.01	0.1	0.04	0.02
WS2	0.03	0.02	0.03	0.03	0.01
BRC1	0.01	0.01	0.1	0.03	0.03
Fibrobacteres	0.01	0	0.02	0.01	0.01
Lentisphaerae	0.02	0	0.01	0.01	0.01

Deferribacteres	0.00	0	0.02	0.01	0.01
BJ-169	0.02	0	0	0.01	0.01
Spirochaetae	0	0	0.01	0.005	0.01
FCPU426	0	0	0.01	0.002	0.004
Gracilibacteria	0	0	0.005	0.002	0.003
SR1_Absconditabacteria	0	0	0.005	0.002	0.003
unclassified	3.9	3.4	5.0	4.1	0.9

Table S3. Relative abundance (%) of the phylum Actinobacteria at different taxonomic levels identified in the three moonmilk deposits in the "Grotte des Collemboles".

		COL1	COL3	COL4	Mean
**Class**	Thermoleophilia	0.0056	0.008	0.023	0.01
**Order**	Gaiellales	0.0056	0.008	0.023	0.01
**Family**	Gaiellaceae	0.0021	0.005	0.015	0.007
	uncultured	0.0035	0.003	0.008	0.005
**Class**	Acidimicrobiia	26.72	9.77	55.32	30.6
**Order**	Acidimicrobiales	26.72	9.77	55.32	30.6
**Family**	uncultured	24.77	8.56	51.6	28.3
	Acidimicrobiaceae	1.16	0.67	2.22	1.4
	Iamiaceae	0.79	0.54	1.5	0.9
**Class**	Actinobacteria	72.75	90	42.98	68.6
**Order**	Pseudonocardiales	14.79	75.3	28.55	39.5
	Corynebacteriales	41	10.09	4.23	18.4
	Streptomycetales	5.37	0.76	3.01	3.0
	Micrococcales	3.76	2.14	2.18	2.7
	Frankiales	1.39	0.46	1.97	1.3
	Propionibacteriales	2.03	0.25	0.48	0.9
	Micromonosporales	1.44	0.52	0.58	0.8
	Streptosporangiales	1.98	0.24	0.1	0.8
	unclasiffied	0.38	0.04	0.96	0.5
	Geodermatophilales	0.09	0.14	0.78	0.3
	Nakamurellales	0.29	0.07	0.07	0.1
	Actinomycetales	0.17	0.001	0.004	0.1
	Glycomycetales	0.02	0	0.04	0.02
	Kineosporiales	0.03	0	0.01	0.01
	Acidothermales	0	0	0.02	0.007

	Catenulisporales	0	0	0.001	0.0003
**Family**	Pseudonocardiaceae	14.79	75.3	28.55	39.5
	Nocardiaceae	39.5	9.94	3.84	17.8
	Streptomycetaceae	5.37	0.76	3.01	3.0
	Micrococcaceae	3.2	1.92	1.84	2.3
	Sporichthyaceae	0.57	0.33	1.76	0.9
	Micromonosporaceae	1.44	0.52	0.58	0.8
	Streptosporangiaceae	1.98	0.24	0.1	0.8
	Propionibacteriaceae	1.65	0.2	0.25	0.7
	Mycobacteriaceae	1.11	0.08	0.35	0.5
	unclassified	0.31	0.04	0.95	0.4
	Microbacteriaceae	0.55	0.19	0.32	0.4
	Geodermatophilaceae	0.09	0.14	0.78	0.3
	Frankiaceae	0.75	0.06	0.1	0.3
	Nocardioidaceae	0.39	0.05	0.23	0.2
	Nakamurellaceae	0.29	0.07	0.07	0.1
	Corynebacteriaceae	0.3	0.05	0.01	0.1
	uncultured	0.16	0.02	0.06	0.08
	Cryptosporangiaceae	0.08	0.06	0.07	0.07
	Actinomycetaceae	0.17	0	0	0.06
	Glycomycetaceae	0.02	0	0.04	0.02
	Kineosporiaceae	0.03	0	0.01	0.01
	Acidothermaceae	0	0	0.02	0.007
	Promicromonosporaceae	0	0.01	0.01	0.007
	Dermacoccaceae	0	0	0.01	0.003
	Gordoniaceae	0	0.01	0	0.003
	Intrasporangiaceae	0	0.01	0	0.003
	Actinospicaceae	0	0	0.001	0.0003
