# Supplementary figures and images for: High-Throughput Sequencing Analysis of the Actinobacterial Spatial Diversity in Moonmilk Deposits
Source: Antibiotics (Basel). 2018 Mar 21;7(2):27. doi: 10.3390/antibiotics7020027 (PMC6023079; doi:10.3390/antibiotics7020027)

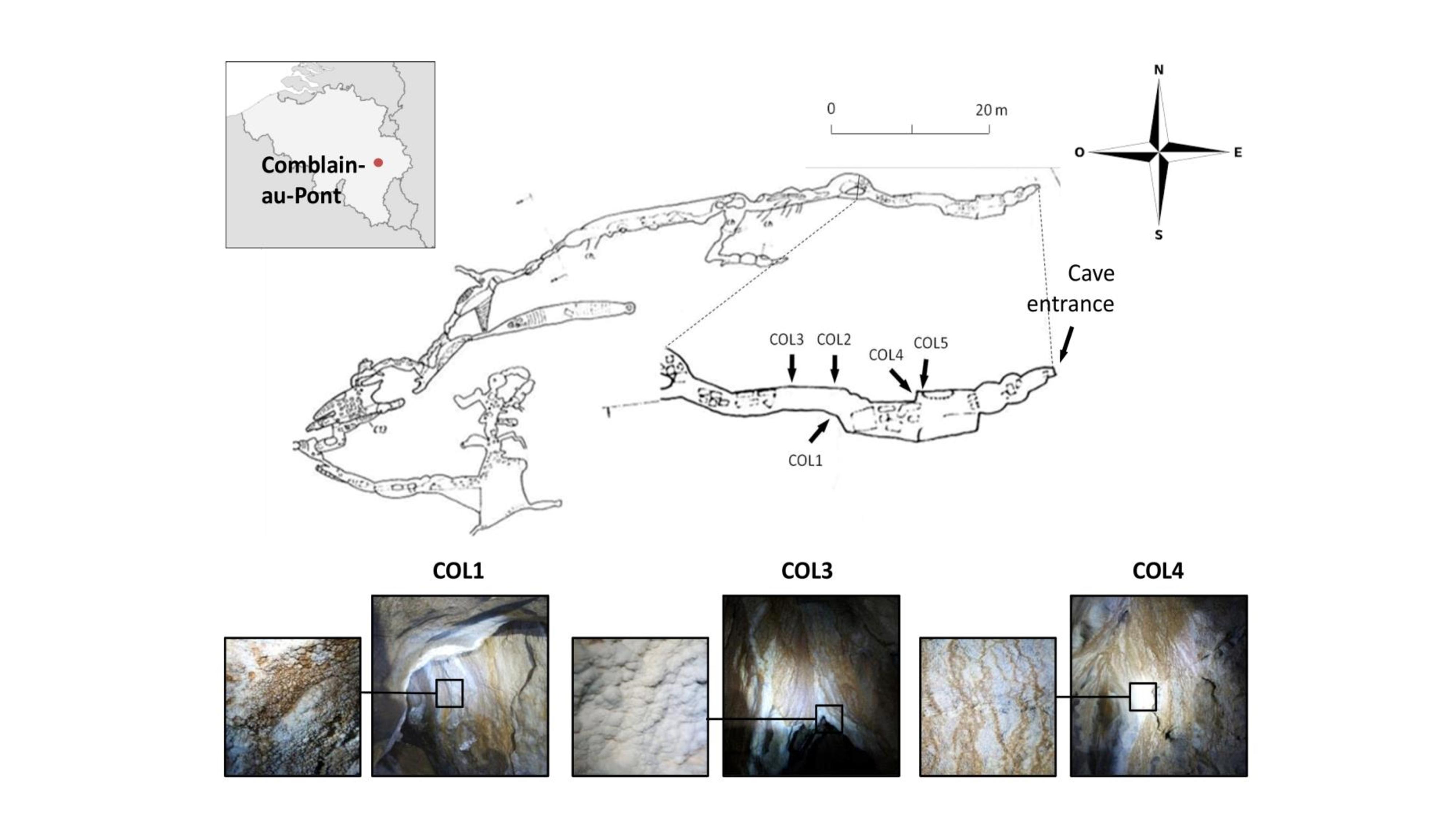

Supplement: Supplementary file 1 [file antibiotics-07-00027-s001.zip › Figure S1.jpg]

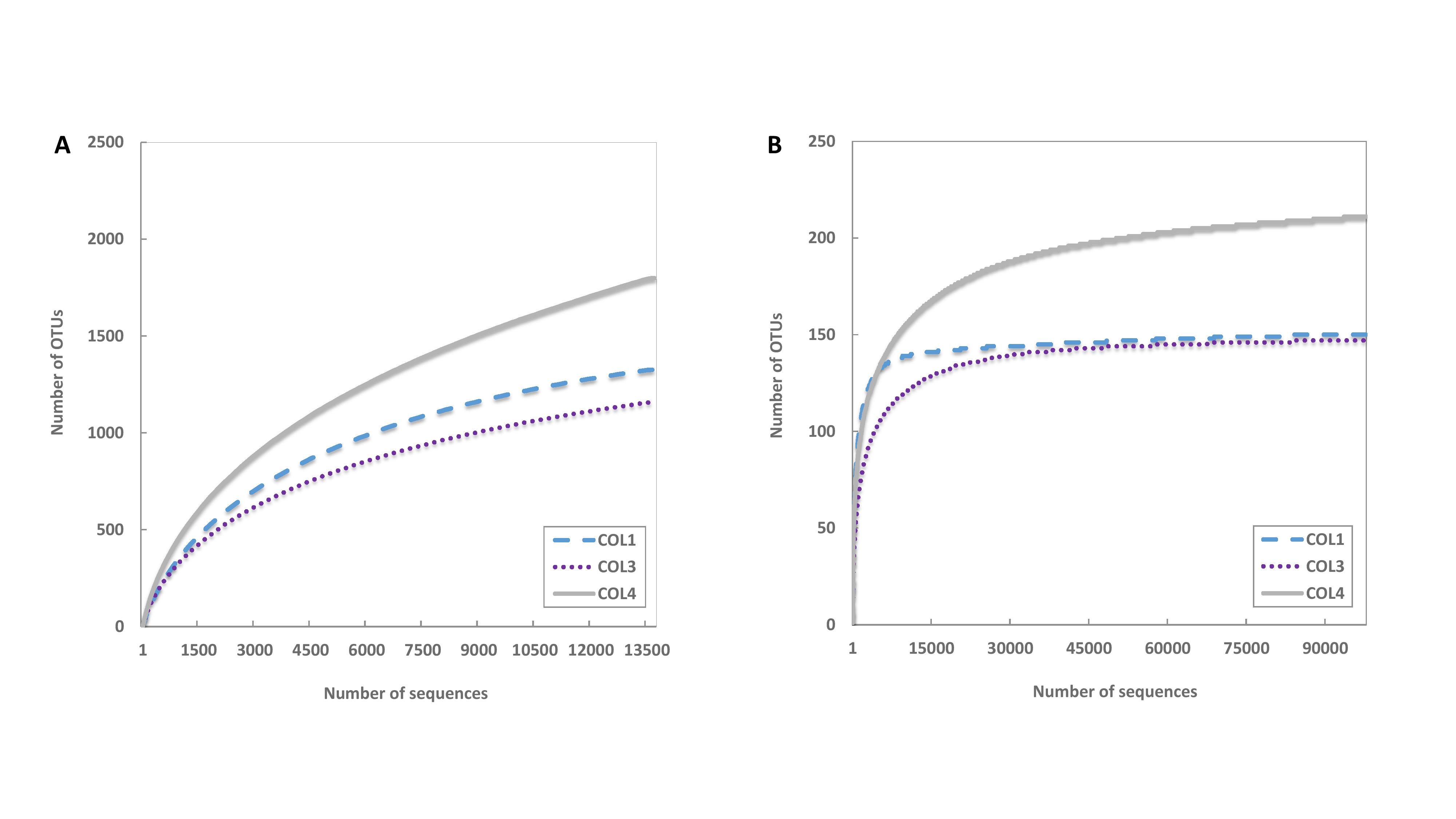

Supplement: Supplementary file 1 [file antibiotics-07-00027-s001.zip › Figure S2.jpg]

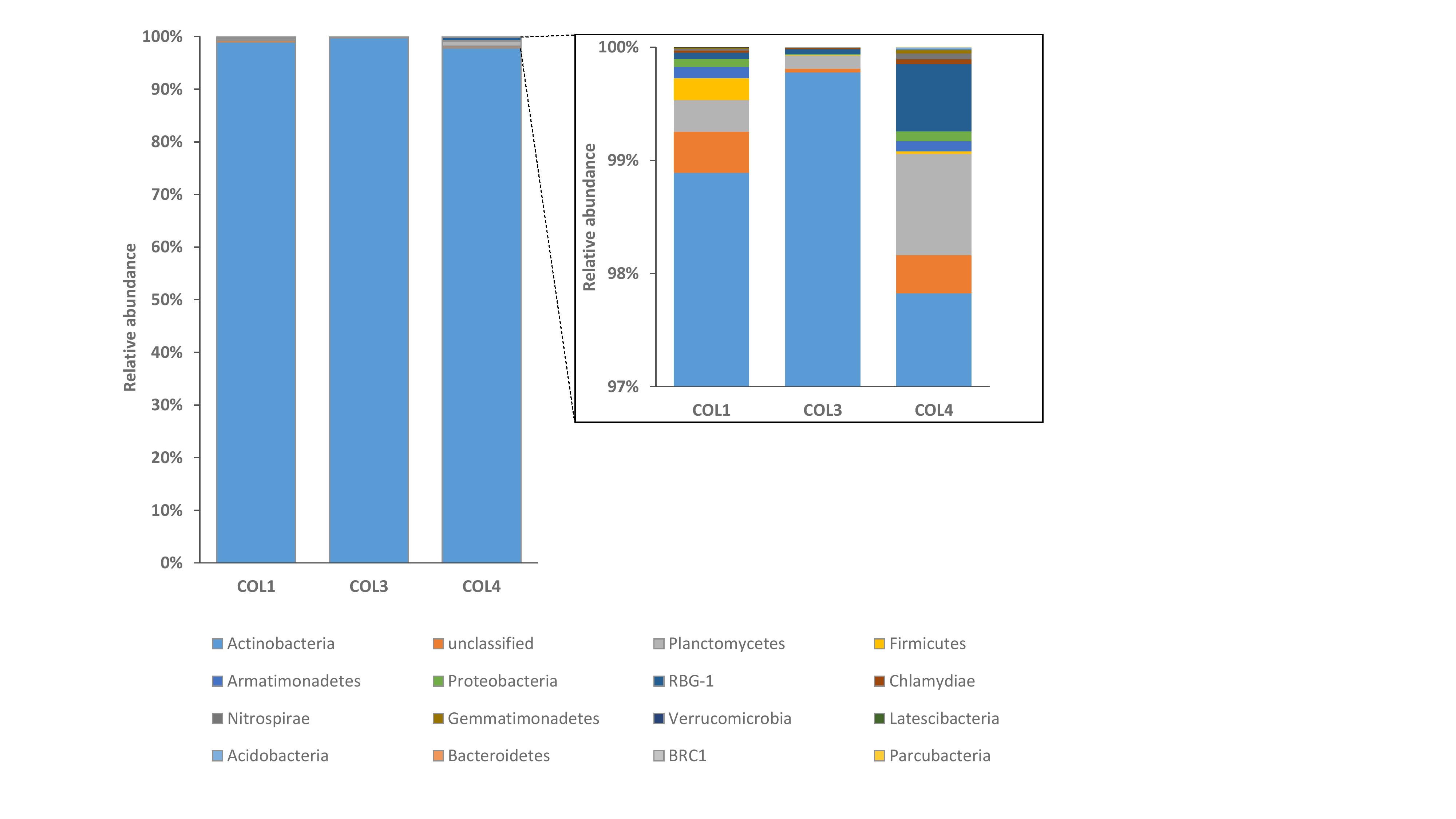

Supplement: Supplementary file 1 [file antibiotics-07-00027-s001.zip › Figure S3.jpg]
